# Supplementary material for: Template plasmids optimized for deletion of multiple genes in yeast Saccharomyces cerevisiae
Source: Microbiol Spectr. 2024 Oct 10;12(11):e01320-24. doi: 10.1128/spectrum.01320-24 (PMC11537097; doi:10.1128/spectrum.01320-24)
Supplement: Supplemental figure and tables — Fig. S1; Tables S1 to S3. [file spectrum.01320-24-s0001.pdf]

A

Expression levels of *S.c.* genes

Gene	Median protein molecules per cell (x1000)
TRP1	2.5
BBC1	10
URA3	26
RPP1B	120
GPM1	180
SSA1	250
TEF1	530
TEF2	530
PGK1	535
FBA1	730
ENO2	730
TDH3	746

B

Homologous marker genes in *S.p.* and *S.c.*

S.p.	*S.c.*
ura4	*URA3*
trp1	*TRP1 + TRP3*
his5	*HIS3*
leu1	*LEU2*

C

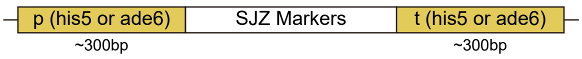

D

Gene knockout feasiblity and fidelity

Case	Intended Change	Verification Method	Correct / Total	Correct rate
C1	*his5* → *his5Δ::nat*	His$^-$ plate selection	14 / 15	93%
C2	*his5* → *his5Δ::hph*	His$^-$ plate selection	15 / 15	100%
C3	*his5* → *his5Δ::kan*	His$^-$ plate selection	8 / 8	100%
C4	*his5* → *his5Δ::LEU2*	PCR	10 / 15	73%
C5	*his5Δ::nat* → *his5*	PCR	7 / 15	47%
C6	*his5* → *his5Δ::URA3*	(Very slow growth on selection plate)		
C7	*ade6* → *ade6Δ::amdS*	(Lawn growth on selection plate)		

E

Case C1: *his5Δ::nat*

Medium NAT+ His-

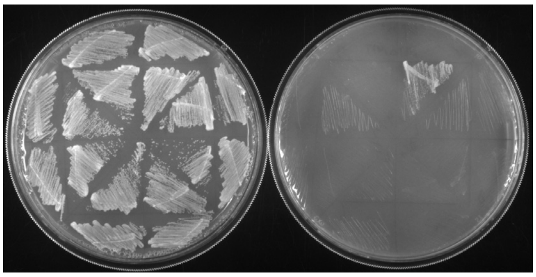

F

Case C2: *his5Δ::hph*

Medium HYM+ His-

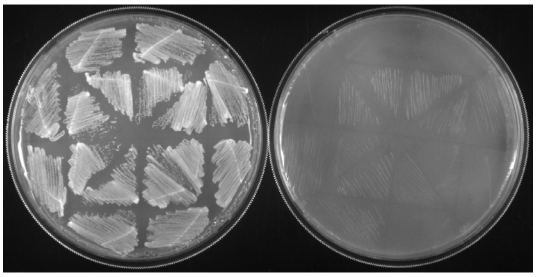

G

Case C3: *his5Δ::kan*

Medium G418+ His-

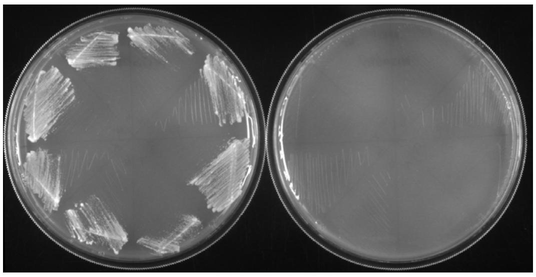

H

Case C4: *his5Δ::LEU2*

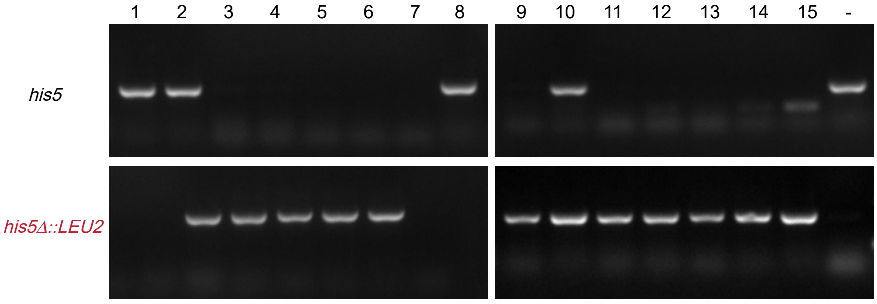

I

Case C5: *his5Δ::nat* → *his5*

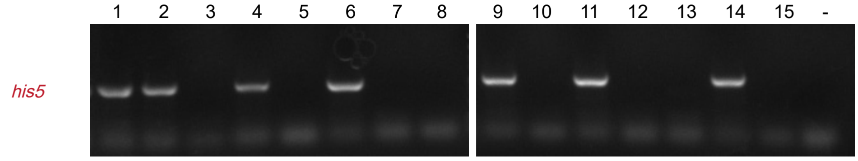

Figure S1. Five of SJZ templates can be used for gene knockout in *S.p.*

A. List of S.c. genes and their expression levels. Data based on (8).

B. Homologous auxotrophic markers in *S.p.* and *S.c.*.

C. Design of gene knockout cassettes for use in S.p.. About 300 bp of homologues targeting sequences flank the selection markers. The flanking sequences target either *his5* or *ade6*.

D-H. Feasibility of using SJZ series templates in *S.p.*. DNA cassettes as outlined in C were tested. In six cases, cassettes containing *nat*, *hph*, *kan*, *LEU2*, *URA3*, and *amdS* markers were used to knockout a wild type gene, either *his5* or *ade6*. In case C5, a cassette containing *his5* was used to knockout a *nat* marker in *his5Δ*::*nat* (constructed in C1), converting the locus to contain *his5* ORF flanked by *S.c.* promoter/terminator in the inner layer, and endogenous *S.p.* promoter/terminator in the outer layer. In case C1, C2, and C3, a His- dropout plate was used to verify the loss of *his5*. In case C4 and C5, colony PCR was used for verification. **D.** Summary of test cases. **E-G.** Loss of *his5* shown by His- selection plates for case C1-C3. **H-I.** Agarose electrophoresis results of colony PCRs for case C4 and C5.

Table S1 Construction of Plasmids

**Plasmid**	Parental Vector 1	Inserted Fragment 2
Inserted Fragment 3	Inserted Fragment 4	Restriction Site
Primers 5'-3'		
**pSJZ1**	pUG72	D-MSC-R (synthesized)
null	null	null
F1: TTTCCATAGGCTCCGCCCCC;		
R1: GACGTCCGCGGAACCCCTATTTGTTTATTTTTCTAAATAC; (Template: pUG72)		
F2: AATAGGGGTTCCGCGGACGTCATTTAGGTGACACTATAGAGCGGCCGC;		
R2: CGGAGCCTATGGAAAGTTAACTAATACGACTCACTATAGGCCGCGGG; (Template: D-MSC-R)		
**pSJZ2**	pSJZ1	null
null	null	null
F1: TCGACGGATCGGTGACGGaGCTGGcTTAATTAAtCCTAGGTGAGGCGCGCCTAGGG;		
R1: CCGTCACCGATCCGTCGACCTGCtGCGTACGAAGCTTCAGCTGGCGGCCGCTCTATAG; (Template: pSJZ1)		
**pSJZ4**	pSJZ1	null
null	null	null
F1: GTCGACGtATtGGaGACGGcGCaGGaTTAATTAAtCCTAGGTGAGGCGCGCCTAGGG;		
R1: CGTCtCCaATaCGTCGACCgGCgGCGTACGAtGCTTCAGCTGGCGGCCGCTCTATAGTG; (Template: pSJZ1)		
**pSJZ5**	pSJZ1	null
null	null	null
F1: GGTCGACGtATaGGaGAtGGTGCaGGaTTAATTAACCCTAGGTGAGGCGCGCCTAGGG;		
R1: CaTCtCCtATaCGTCGACCTGCgGCGTACGAAGCTTCAGCTGGCGGCCGCTCTATAGTG; (Template: pSJZ1)		
**pSJZ6**	pSJZ1	null
null	null	null
F1: GTCGACGaATaGGcGAtGGcGCTGGTTTAATTAAtCCTAGGTGAGGCGCGCCTAGGG;		
R1: CaTCgCCtATtCGTCGACCaGCtGCGTACGAgGCTTCAGCTGGCGGCCGCTCTATAGTG; (Template: pSJZ1)		
**pSJZ7**	pSJZ1	null
null	null	null
F1: tGGTCGACGGATCGGTGACGGTGCcGGcTTAATTAACCCTAGGTGAGGCGCGCCTAGGG;		
R1: TCACCGATCCGTCGACCaGCgGCGTACGAtGCTTCAGCTGGCGGCCGCTCTATAGTGTC; (Template: pSJZ1)		
**pSJZ9**	pSJZ1	null
null	null	null
F1: GTCGACGtATtGGTGAtGGaGCaGGTTTAATTAACCCTAGGTGAGGCGCGCCTAGGG;		
R1: CaTCACCaATaCGTCGACCaGCAGCGTACGAgGCTTCAGCTGGCGGCCGCTCTATAGTG; (Template: pSJZ1)		
**pSJZ10**	pSJZ1	null
null	null	null
F1: TCGACGaATtGGaGACGGTGCTGGaTTAATTAAtCCTAGGTGAGGCGCGCCTAGGG;		
R1: ACCGTCtCCaATtCGTCGACCtGCgGCGTACGAtGCTTCAGCTGGCGGCCGCTCTATAG; (Template: pSJZ1)		

**Plasmid**	Parental Vector 1	Inserted Fragment 2
Inserted Fragment 3	Inserted Fragment 4	Restriction Site
Primers 5'-3'		
**pSJZ1-URA3**	pSJZ1	pRPP1B
URA3 C.a.	tSSA1	SacI
F2: CCTAGGGATAACAGGGTAATCCTTTCAAAGTGATTGGTCATTGTTAAGGC;		
R2: GACTGTCATTTTCTTCTTAGTTGTTTGATTTCTTTGTTAGTCCAAG; (Template: pRPP1B from genome)		
F3: TAAGAAGAAAATGACAGTCAACACTAAGACCTATAGTGAGAG;		
R3: CCAATTGGCTTATAATTGGCCAGTCTTTTTCAAATAAGCATTCCAACC; (Template: URA3 C.a. from pKT209)		
F4: CAATTATAAGCCAATTGGTGCGGCAATTG;		
R4: AGGCCACTAGTGGATCGAGCTCACAGGAAGACAAAGCATGCGAGAGG; (Template: tSSA1 from genome)		
**pSJZ2-TRP1**	pSJZ2	pBBC1
TRP K.l.	tSSA2	SacI
F2: CCTAGGGATAACAGGGTAATTTTGAGTTATTCATTTCCAGCACTTCCAGG;		
R2: TAACGAGCATCCTTAGTTTGGGTAAGACTGTGTTTACTTC; (Template: pBBC1 from genome)		
F3: CAAACTAAGGATGCTCGTTAAAGTGTGTGGTTTGC;		
R3: TCGCTAAGCCTATTGAGAGGCCTGCTGGATGAATAGC; (Template: TRP K.l. from pUG76)		
F4: TCTCAATAGGCTTAGCGATCGCCCTGTAAAATATCCG;		
R4: GGCCACTAGTGGATCGAGCTCGCGTATATACACATTGTATACTGTTGATATTATCAAG; (Template: tSSA2 from genome)		
**pSJZ4-nat**	pSJZ1	pFBA1
nat	tTDH3	SacI
F2: GCCTAGGGATAACAGGGTAATTGGGTCATTACGTAAATAATGATAGGAATGGG;		
R2: tggtacccatTTTGAATATGTATTACTTGGTTATGGTTATATATGAC; (Template: pFBA1 from genome)		
F3: CATATTCAAAatgggtaccactcttgacgacac;		
R3: TAAATTCACttaggggcagggcatgctcatg; (Template: NAT from pUG74)		
F4: cctgcccctaaGTGAATTTACTTTAAATCTTGCATTTAAATAAATTTTCTTTTTATAGC;		
R4: GGCCACTAGTGGATCGAGCTCATCCTGGCGGAAAAAATTCATTTGTAAAC; (Template: tTDH3 from genome)		
**pSJZ5-hph**	pSJZ5	pSSA1
hph	tFBA1	SacI
F2: CCTAGGGATAACAGGGTAATAGAAAAAAAATTTCCTTGGTTGAAAATGGCGG;		
R2: TTTTACCCATATTATCTGTTATTTACTTGAATTTTTGTTTCTTGTAATAC; (Template: pSSA1 from genome)		
F3: AACAGATAATATGGGTAAAAAGCCTGAACTCACCG;		
R3: TTGAATTAACTTATTCCTTTGCCCTCGGACGAG; (Template: HPH from pUG75)		
F4: AAAGGAATAAGTTAATTCAAATTAATTGATATAGTTTTTTAATGAGTATTGAATCTG;		
R4: GGCCACTAGTGGATCGAGCTCAATGAGCTATCAAAAACGATAGATCG; (Template: tFBA1 from genome)		
**pSJZ6-his5**	pSJZ6	pTEF1
his S.p.	tAHP1	SacI
F2: GCCTAGGGATAACAGGGTAATAAGCAACAGGCGCGTTGGAC;		
R2: TCCTACCCATTTTGTAATTAAAACTTAGATTAGATTGCTATGCTTTCTTTC; (Template: pTEF1 from genome)		
F3: TAATTACAAAATGGGTAGGAGGGCTTTTGTAGAAAG;		
R3: TACATAGCATTTACAACACTCCCTTCGTGCTTGG; (Template: HIS5 S.p. from pUG27)		
F4: AGTGTTGTAAATGCTATGTAATAGACAATAAAACCATG; (Template: tAHP1 from genome)		
R4: GGCCACTAGTGGATCGAGCTCGGCATTTAACAAATATATAACAATTGGGATTTAGTAG;		

**pSJZ7-kan**	pSJZ7	pGPM1
kan	tPDC1	SacI
F2: CCTAGGGATAACAGGGTAATGCACGCGCGATGGTGCTAAG;		
R2: CCTTACCCATTATTGTAATATGTGTGTTTGTTTGGATTATTAAGAAG; (Template: pGPM1 from genome)		
F3: TATTACAATAATGGGTAAGGAAAAGACTCACGTTTCG;		
R3: ATTAAATCGCTTAGAAAAACTCATCGAGCATCAAATGAAACTGC; (Template: KAN from pUG6)		
F4: GTTTTTCTAAGCGATTTAATCTCTAATTATTAGTTAAAGTTTTATAAGC;		
R4: GGCCACTAGTGGATCGAGCTCTGTTCCTTAATCAAGGATACCTCTTTTTTTTTCCTTGG; (Template: tPDC1 from genome)		
**pSJZ9-amdS**	pSJZ9	pENO2
amdS	tTEF1	SacI
F2: GCCTAGGGATAACAGGGTAATCGACGTGCACCAACTTGCGGAAAG;		
R2: ATTGTGGCATTATTATTGTATGTTATAGTATTAGTTGCTTGGTGTTATGAAAG; (Template: pENO2 from genome)		
F3: TACAATAATAATGCCACAATCTTGGGAAGAATTGG;		
R3: ATCAATCTCCTTATGGAGTAACAACGTTACCCAACAAC; (Template: amdS from pUG-amdSYM)		
F4: TACTCCATAAGGAGATTGATAAGACTTTTCTAGTTGCATATC;		
R4: AGGCCACTAGTGGATCGAGCTCAAAAGACCAAACGGTGACGTTAAGAG; (Template: tTEF1 from genome)		
**pSJZ10-LEU2**	pSJZ10	pLEU2-LEU2 K.l.
tTEF2	null	SacI
F2: GCCTAGGGATAACAGGGTAATCTCGTCAAATTTCAAACGCTGCCAC;		
R2: ATTACTCTTATTAAGCCAAGATTTCCTTGACAGCC; (Template: pLEU2-LEU2 K.l. from pUG73)		
F3: CTTGGCTTAATAAGAGTAATAATTATTGCTTCCATATAATATTTTTATATACCTC;		
R3: AGGCCACTAGTGGATCGAGCTCACGCTGACATGGTTTCTTTAGGTTTG; (Template: tTEF2 from genome)		
**pSJZ-his5D-nat**	pSJZ1	p his5
nat	t his	null
F2: acaaacgtccgattccataCCGCGGCCTATAGTGAGTCGTATTAG;		
R2: tggttattcatattcaacgGCTAGCGATGTTAATTAACCCAGGAATCC; (Template: pSJZ1)		
F3: cgttgaatatgaataaccaatttcagcg;		
R3: CTAGGCGCGCCTCACCTAGGcgtgatgcaaaactactcttttcaattag; (Template: p his5 from genome)		
F4: CCTAGGTGAGGCGCGCCTAG;		
R4: GCATAGGCCACTAGTGGATCGAGC; (Template: nat from pSJZ4-nat)		
F5: GCTCGATCCACTAGTGGCCTATGCagatgaattggattatgtcaggaaaagaacg ;		
R5: tatggaatcggacgtttgtgcatc; (Template: t his5 from genome)		
**pSJZ-his5D-hph**	pSJZ-his5D-nat	hph
null	null	null
F2: GCTCGATCCACTAGTGGCCTATGCagatgaattggattatgtcaggaaaagaacg;		
R2: CTAGGCGCGCCTCACCTAGGcgtgatgcaaaactactcttttcaattag; (Template: pSJZ-his5D-nat)		
F3: CCTAGGTGAGGCGCGCCTAG;		
R3: GCATAGGCCACTAGTGGATCGAGC; (Template: hph from pSJZ5-hph)		

**pSJZ-his5D-kan**	pSJZ-his5D-nat	kan
null	null	null
F2: GCTCGATCCACTAGTGGCCTATGCagatgaattggattatgtcaggaaaagaacg;		
R2: CTAGGCGCGCCTCACCTAGGcgtgatgcaaaactactcttttcaattag; (Template: pSJZ-his5D-nat)		
F3: CCTAGGTGAGGCGCGCCTAG;		
R3: GCATAGGCCACTAGTGGATCGAGC; (Template: kan from pSJZ7-kan)		
**pSJZ-his5D-LEU2**	pSJZ-his5D-nat	LEU2
null	null	null
F2: GCTCGATCCACTAGTGGCCTATGCagatgaattggattatgtcaggaaaagaacg;		
R2: CTAGGCGCGCCTCACCTAGGcgtgatgcaaaactactcttttcaattag; (Template: pSJZ-his5D-nat)		
F3: CCTAGGTGAGGCGCGCCTAG;		
R3: GCATAGGCCACTAGTGGATCGAGC; (Template: LEU2 from pSJZ10-LEU2)		
**pSJZ-his5D-his5**	pSJZ-his5D-nat	his5
null	null	null
F2: GCTCGATCCACTAGTGGCCTATGCagatgaattggattatgtcaggaaaagaacg;		
R2: CTAGGCGCGCCTCACCTAGGcgtgatgcaaaactactcttttcaattag; (Template: pSJZ-his5D-nat)		
F3: CCTAGGTGAGGCGCGCCTAG;		
R3: GCATAGGCCACTAGTGGATCGAGC; (Template: his5 from pSJZ6-his5)		
**pSJZ-his5D-URA3**	pSJZ-his5D-nat	URA3
null	null	null
F2: GCTCGATCCACTAGTGGCCTATGCagatgaattggattatgtcaggaaaagaacg;		
R2: CTAGGCGCGCCTCACCTAGGcgtgatgcaaaactactcttttcaattag; (Template: pSJZ-his5D-nat)		
F3: CCTAGGTGAGGCGCGCCTAG;		
R3: GCATAGGCCACTAGTGGATCGAGC; (Template: URA3 from pSJZ1-URA3)		
**pSJZ-ade6D-amdS**	pSJZ1	p ade6
amdS	t ade6	null
F2: acaaacgtccgattccataCCGCGGCCTATAGTGAGTCGTATTAG;		
R2: tggttattcatattcaacgGCTAGCGATGTTAATTAACCCAGGAATCC; (Template: pSJZ1)		
F3: agcctggtgcagtataaggtataacg;		
R3: CCTAGGCGCGCCTCACCTAGGattcgatgaagtatgtatataccttggcag; (Template: p ade6 from genome)		
F4: CCTAGGTGAGGCGCGCCTAG;		
R4: GCATAGGCCACTAGTGGATCGAGC; (Template: amdS from pSJZ9-amdS)		
F5: CTCGATCCACTAGTGGCCTATGCtaggcgaccatagacataactgttaaatg ;		
R5: tatgcgttaatttaattgggaacatggtcaac; (Template: t ade6 from genome)		

Table S2 Primers for Knockout and Verification

**Name**	**Sequence 5'-3'**
**Knockout Primers**	
atg11-T1/D1	GTTGTTCGGAAAGTACTTCTTTTATTTTCTTTTATACATCGCTTCGTACGCTGCAGGTC
atg11- D5	GTTGTTCGGAAAGTACTTCTTTTATTTTCTTTTATACATCGCTTCGTACGCCGCAGGTC
atg11- LRS	TAAAATCTTGTCATTTGTGACAAACGTTTAGCACTGTTCAGCATAGGCCACTAGTGGAT
atg17-T1/D1	ATTCGATACT GCGAGGATAT TATCAACGTA TTTAACACCTGCTTCGTACGCTGCAGGTC
atg17- D7	ATTCGATACTGCGAGGATATTATCAACGTATTTAACACCTGCATCGTACGCCGCTGGTC
atg17 LRS	ATTGAATCTTTGTACCGTATCCTTTTTTTCCTTTTTTCTAGCATAGGCCACTAGTGGAT
atg23-T1/D1	GTTGTTCTATAAGGTAACAAAATAAAGTGAAGAAGTAAATGCTTCGTACGCTGCAGGTC
atg23- D7	GTTGTTCTATAAGGTAACAAAATAAAGTGAAGAAGTAAATGCATCGTACGCCGCTGGTC
atg23- LRS	TTACATTATCCTCATGGCTACTCTAGCTATTTGCATTTCAGCATAGGCCACTAGTGGAT
atg27- D2	TCAATCGATGCGATAGATAAAGGTAAGGAAAGCTTTCACGGCTTCGTACGCAGCAGGTC
atg27- LRS	GTTGCAAAAATATCGAATTGTAAGCCAGTAAACTTATTTAGCATAGGCCACTAGTGGAT
atg29-T1/D1	TACATAATTGTACTGCTGTGCATTTTCCTACTTGACTTTCGCTTCGTACGCTGCAGGTC
atg29-D2	TACATAATTGTACTGCTGTGCATTTTCCTACTTGACTTTCGCTTCGTACGCAGCAGGTC
atg29-D4	TACATAATTGTACTGCTGTGCATTTTCCTACTTGACTTTCGCATCGTACGCCGCCGGTC
atg29-D5	TACATAATTGTACTGCTGTGCATTTTCCTACTTGACTTTCGCTTCGTACGCCGCAGGTC
atg29-D6	TACATAATTGTACTGCTGTGCATTTTCCTACTTGACTTTCGCCTCGTACGCAGCTGGTC
atg29-D7	TACATAATTGTACTGCTGTGCATTTTCCTACTTGACTTTCGCATCGTACGCCGCTGGTC
atg29-D9	TACATAATTGTACTGCTGTGCATTTTCCTACTTGACTTTCGCCTCGTACGCTGCTGGTC
atg29-D10	TACATAATTGTACTGCTGTGCATTTTCCTACTTGACTTTC GCaTCGTACGCcGCaGGTC
atg29-LRS	CAGTTGGTTTTTTGATTGTGCTTGTGAAAGATGTAAATCAGCATAGGCCACTAGTGGAT
ymr1- D6	CTAAAAAGAGGGAAAAGAAATAGTATACCATTCCGCAAAGGCCTCGTACGCAGCTGGTC
ymr1-LRS	CTAACTCACTTATTTGTCTTCTTTGCCGTTAACCAGATCA GCATAGGCCACTAGTGGAT
his5- pF	cgttgaatatgaataaccaatttcagcg
his5- tR	tatggaatcggacgtttgtgcatc
ade6- pF	agcctggtgcagtataaggtataacg
ade6- tR	tatgcgttaatttaattgggaacatggtcaac
**Verification Primers**	
ATG1-VF	AACCGCTCGGCTCTGATTTC
ATG11-VF	CTTCGTTACGTATGTCCAATGGC
ATG11-VIR	CGGGAGGAATCTTCCATTTCC
ATG17-VF	CTGTGTTGGAGTCCGAATCCTCC
ATG17-VIR	AGCTTTGTCCCAATGCCTTCC
ATG21-VF	TTCACACTCGCACTCGGATG
ATG23-VF	GTAAGCTCTGGCGTTTAGGTGC
ATG23-VIR	GTGATATCCTTCCGGATGCTTGTC
ATG27-VF	GGCCATGTATGGGTGAGAATGTCC
ATG27-VIR	GGGCAAGATGACATCTGTTAAACCAC
ATG29-VF	TCTAGACGCACTGCACTCGTC
ATG29-VIR	CCTGTCTTTCCGTGGTCAGTG
ATG31-VF	TCGCCTTTACACGGCGAAG
VPS38-VF	GGTAGAGCTCCACAACAAGTTGAG

Name	Sequence 5'-3'
URA3 C.a.-VIR	CGtTGTGCTACTGGTGAGGC
URA3 K.L.-VIR	CCATTGAAAGCATTGGCAGA
TRP1 K.l.-VIF	CGATCGGTGTCGATGTAAGTGGAGG
TRP1 K.l.-VIR	TCAGCACCATCATCCACAGCAG
NAT-VIR	ACTGGTGCGGTACCGGTAAG
HPH-VIR	CATCAGGTCGGAGACGCTGTCG
HIS5 S.p.-VIR	GCCTGCATGTTTAGCCAGTGC
G418-VIR	GCGGCCTCGAAACGTGAGTC
amdSYM-VIR	CAATCTAGCTCTCTTGTCAGCAGCC
LEU2 K.l.-VIR	CGTCAGTAACTTCTTTACCGACGTG
his5- VF	gggtcctcaattcgatgttgcag
his5- VR	gtcacatacgttgaacagttgaattcg
ade6- VF	acgcacattgaaacatggacgac
ade6- VR	cttttgctgcaatcagcgcc
ade6- VIF	gccaaaatgccggtattttagcc
ade6- VIR	cccaattgaccacctccaagg

Table S3 List of Strains

Name	Genotype	Notes
S.c.		
BY4742	*MATα his3Δ1 leu2Δ lys2Δ ura3Δ*	PMID: 9483801
TN124	*MATa leu2-3,112 trp1 ura3-52 pho8::pho8Δ60 pho13::LEU2*	PMID: 7741731
YJZ757	*TN124 met17Δ his3Δ*	This work
YJZ967	*TN124 met17Δ his3Δ atg29Δ::his5 (UG27) atg31Δ::nat (UG74)*	This work
YJZ3336	*TN124 ura3::URA3 atg21Δ::nat (UG74)*	This work
YJZ3422	*TN124 atg1Δ::kan (UG6) vps38Δ::hph (UG75)*	This work
YS127	*TN124 atg27Δ::TRP1 (UG76)*	This work
YS131	*TN124 atg31Δ::TRP1 (UG76)*	This work
YJZ4518	*TN124 ura3::URA3 atg21Δ::nat (UG74) atg29Δ::hph (UG75)*	Fig.2
YJZ4513	*TN124 met17Δ his3Δ atg29Δ::his5 (UG27) atg31Δ::nat (UG74) atg17Δ::kan (UG6)*	Fig.2
YJZ4512	*TN124 met17Δ his3Δ atg29Δ::his5 (UG27) atg31Δ::nat (UG74) atg11Δ::hph (UG75)*	Fig.2
YJZ4516	*TN124 atg1Δ::kan (UG6) vps38Δ::hph (UG75) atg29Δ::nat (UG74)*	Fig.2
YJZ4505	*TN124 atg27Δ::TRP1 (UG76) atg23Δ::URA3 (UG72)*	Fig.2
YJZ4507	*TN124 atg31Δ::TRP1 (UG76) atg29Δ::URA3 (UG72)*	Fig.2
YJZ4482	*TN124 met17Δ his3Δ atg29Δ::URA3 (SJZ1-URA3)*	Fig.4
YJZ4483	*TN124 met17Δ his3Δ atg29Δ::TRP1 (SJZ2-TRP1)*	Fig.4
YJZ4485	*TN124 met17Δ his3Δ atg29Δ::nat (SJZ4-NAT)*	Fig.4
YJZ4484	*TN124 met17Δ his3Δ atg29Δ::hph (SJZ5-HPH)*	Fig.4
YJZ4486	*TN124 met17Δ his3Δ atg29Δ::his5 (SJZ6-HIS5)*	Fig.4
YJZ4487	*TN124 met17Δ his3Δ atg29Δ::kan (SJZ7-KAN)*	Fig.4
YJZ4488	*TN124 met17Δ his3Δ atg29Δ::amdS (SJZ9-amdS)*	Fig.4
YJZ4489	*BY4742 atg29Δ::LEU2 (SJZ10-LEU2)*	Fig.4
YJZ4491	*TN124 met17Δ his3Δ atg23Δ::URA3 (SJZ1-URA3)*	Fig.4
YJZ4492	*TN124 met17Δ his3Δ atg27Δ::TRP1 (SJZ2-TRP1)*	Fig.4
YJZ4450	*TN124 met17Δ his3Δ atg11Δ::hph (SJZ5-HPH)*	Fig.4
YJZ4502	*TN124 met17Δ his3Δ ymr1Δ::his5 (SJZ6-HIS5)*	Fig.4
YJZ4503	*TN124 met17Δ his3Δ atg23Δ::kan (SJZ7-KAN)*	Fig.4
YJZ4517	*TN124 ura3::URA3 atg21Δ::nat (UG74) atg29Δ::hph (SJZ5-HPH)*	Fig.4
YJZ4514	*TN124 met17Δ his3Δ atg29Δ::his5 (UG27) atg31Δ::nat (UG74) atg17Δ::kan (SJZ7-KAN)*	Fig.4
YJZ4511	*TN124 met17Δ his3Δ atg29Δ::his5 (UG27) atg31Δ::nat (UG74) atg11Δ::hph (SJZ5-HPH)*	Fig.4
YJZ4515	*TN124 atg1Δ::kan (UG6) vps38Δ::hph (UG75) atg29Δ::nat (SJZ4-NAT)*	Fig.4
YJZ4506	*TN124 atg27Δ::TRP1 (UG76) atg23Δ::URA3 (SJZ1-URA3)*	Fig.4
YJZ4508	*TN124 atg31Δ::TRP1 (UG76) atg29Δ::URA3 (SJZ1-URA3)*	Fig.4
YJZ4519	*TN124 met17Δ his3Δ atg29Δ::nat (SJZ4-NAT) atg23Δ::kan (SJZ7-KAN)*	Fig.4
YJZ4520	*TN124 met17Δ his3Δ atg29Δ::nat (SJZ4-NAT) atg23Δ::kan (SJZ7-KAN) atg11Δ::hph (SJZ5-HPH)*	Fig.4
S.p.		
LD331	*h^+*	from Lilin Du lab
DY43884	*h^- ura4-D18 leu1-32 his3-D1*	from Lilin Du lab
YSP001	*LD331 his5Δ::nat*	Fig.S1
YSP002	YSP001 *his5*	Fig.S1

Name	Genotype	Notes
YSP003	*DY43884 his5Δ::LEU2*	Fig.S1
YSP004	*LD331 his5Δ::kan*	Fig.S1
YSP006	*LD331 his5Δ::hph*	Fig.S1
